# Expression Profiles of Neuropeptides, Neurotransmitters, and Their Receptors in Human Keratocytes *In Vitro* and *In Situ*

**DOI:** 10.1371/journal.pone.0134157

**Published:** 2015-07-27

**Authors:** Marta Słoniecka, Sandrine Le Roux, Peter Boman, Berit Byström, Qingjun Zhou, Patrik Danielson

**Affiliations:** 1 Department of Integrative Medical Biology, Umeå University, Umeå, Sweden; 2 Department of Clinical Sciences, Ophthalmology, Umeå University, Umeå, Sweden; 3 Shandong Provincial Key Laboratory of Ophthalmology, Shandong Eye Institute, Qingdao, China; Cedars-Sinai Medical Center; UCLA School of Medicine, UNITED STATES

## Abstract

Keratocytes, the quiescent cells of the corneal stroma, play a crucial role in corneal wound healing. Neuropeptides and neurotransmitters are usually associated with neuronal signaling, but have recently been shown to be produced also by non-neuronal cells and to be involved in many cellular processes. The aim of this study was to assess the endogenous intracellular and secreted levels of the neuropeptides substance P (SP) and neurokinin A (NKA), and of the neurotransmitters acetylcholine (ACh), catecholamines (adrenaline, noradrenaline and dopamine), and glutamate, as well as the expression profiles of their receptors, in human primary keratocytes *in vitro* and in keratocytes of human corneal tissue sections *in situ*. Cultured keratocytes expressed genes encoding for SP and NKA, and for catecholamine and glutamate synthesizing enzymes, as well as genes for neuropeptide, adrenergic and ACh (muscarinic) receptors. Keratocytes in culture produced SP, NKA, catecholamines, ACh, and glutamate, and expressed neurokinin-1 and -2 receptors (NK-1R and NK-2R), dopamine receptor D_2_, muscarinic ACh receptors, and NDMAR1 glutamate receptor. Human corneal sections expressed SP, NKA, NK-1R, NK-2R, receptor D_2_, choline acetyl transferase (ChAT), M_3_, M_4_ and M_5_ muscarinic ACh receptors, glutamate, and NMDAR1, but not catecholamine synthesizing enzyme or the α_1_ and β_2_ adrenoreceptors, nor M_1_ receptor. In addition, expression profiles assumed significant differences between keratocytes from the peripheral cornea as compared to those from the central cornea, as well as differences between keratocytes cultured under various serum concentrations. In conclusion, human keratocytes express an array of neuropeptides and neurotransmitters. The cells furthermore express receptors for neuropeptides/neurotransmitters, which suggests that they are susceptible to stimulation by these substances in the cornea, whether of neuronal or non-neuronal origin. As it has been shown that neuropeptides/neurotransmitters are involved in cell proliferation, migration, and angiogenesis, it is possible that they play a role in corneal wound healing.

## Introduction

The cornea is composed of the outer stratified squamous epithelium, the intermediate stroma, and the inner endothelium.[1] The stroma consists of type I/V collagen fibers and proteoglycan decorin, lumican, keratocan, and osteoglycin/mimecan [2]. Type III collagen is also present in low proportions but it increases during wound healing and inflammation [3]. The keratocytes, located between the collagen lamellae in the stroma, are a population of quiescent, mesenchymal-derived cells [4]. Despite being sparsely arranged in the stroma, keratocytes form an interconnected cellular network through long dendritic processes [5].

Upon injury, the keratocytes may either undergo apoptosis or transdifferentiate into an activated fibroblastic repair phenotype. This fibroblastic phenotype seen in corneal wound healing resembles the phenotype that is seen under culture conditions *in vitro*. Standard culture conditions for corneal stromal cells (10% Fetal Calf Serum) alter the keratocytes from their *in situ* phenotype [6]. Of the total glycosaminoglycans (GAGs) synthesized by corneas in organ culture, 47% are keratan sulfates [7]. However, stromal cells derived from bovine, rabbit and human corneas, cultured under standard conditions, have been reported to produce moderate (15%), little (3%), or no keratan sulfates, respectively [6, 8–10]. Serum-cultured corneal stromal cells also express the fibronectin receptor a5b1 (which is not expressed by keratocytes *in situ*), they have a fibroblastic morphology, and their actin cytoskeleton resembles the one of corneal fibroblasts or even corneal myofibroblasts [11–13]. However, when primary cultures of corneal stromal cells are cultured in serum-free medium, they exhibit a dendritic morphology and extensive dendritic processes [14], and their appearance is similar to keratocytes *in situ* and distinctly different from the fibroblastic or myofibroblastic appearance of keratocytes grown in serum-containing medium [5, 14].

Stromal wound healing consists of three stages: repair, regeneration (proliferation and migration of keratocytes [15]), and remodeling [16], and has been shown to involve a complex interplay between cytokines, growth factors, and chemokines [17]. Based on a number of observations, it is likely that also other signal substances are at play such as neuropeptides [18] and other classical neuronal transmitters [19–21]. To further elucidate the role of such substances in corneal wound healing it is of importance to study their expression profiles in corneal stromal cells *in situ* and *in vitro*. Similarly, the presence of their receptors is also important to delineate, since the influence of neuropeptides/neurotransmitters on primary corneal cells may depend on not only non-neuronal but also neuronal signaling in the cornea.

The cornea is one of the most innervated tissues in the body, containing nerve fibers derived from the trigeminal ganglion. Previous studies have demonstrated that the corneal nerve fibers exert important trophic influences and contribute to the homeostatic maintenance of corneal epithelium [22]. However, the potential role of neuropeptides or neurotransmitters in corneal stromal wound healing remains poorly understood. Studies have confirmed the involvement of nerves, especially nerves secreting neuropeptides, in the processes of diabetic/skin wound healing, including inflammation, epithelialization, and fibrogenesis [23–27].

The neuropeptides substance P (SP) and neurokinin A (NKA) belong to the tachykinin family which have a variety of pharmacological actions both in the central nervous system and in the periphery [28]. SP and its preferred receptor, the neurokinin-1 receptor (NK-1R), have been found to be expressed by a wide range of not only neuronal but also non-neuronal human cells [18, 29] NKA signals through its high affinity receptor, the neurokinin-2 receptor (NK-2R). Acetylcholine (ACh) is regarded as a classical neurotransmitter, and it acts through either nicotinic ACh receptors (nAChRs) or muscarinic ACh receptors (mAChRs) [30]. Catecholamines (dopamine, adrenaline and noradrenaline) constitute a class of chemical neurotransmitters and hormones that occupy key positions in regulation of physiological processes. Adrenaline and noradrenaline act through α (α_1_ and α_2_) and β (β_1_ and β_2_) adrenoceptors (adrenergic receptors) in target cells whereas dopamine acts through its D_1_ (D_1_ and D_5_) and D_2_ (D_2_, D_3_ and D_4_) classes of receptors. Glutamate is a non-essential amino acid, which binds to the N-Methyl-D-aspartic acid receptor (NMDAR) and acts as a major neurotransmitter in the mammalian central nervous system. All these neuropeptides and neurotransmitters are known to exert effects on processes involved in wound healing.

Considering the importance of keratocytes in corneal stromal wound healing, it would be of interest to study the expression profiles of neuropeptides and neurotransmitters in human keratocytes. Therefore, as it has not been documented before and as a basis for further functional studies on the role of neuropeptides/neurotransmitters in corneal wound healing, the present work studied the endogenous intracellular and secreted levels of the tachykinins SP and NKA, and of ACh, catecholamines (adrenaline, noradrenaline and dopamine), and glutamate, as well as the expression profiles of their receptors, in human primary keratocytes *in vitro* and in keratocytes of human corneal tissue sections *in situ*.

## Materials and Methods

### Collection of human corneas

Healthy human corneas from deceased individuals who had chosen, when alive, to donate their corneas post-mortem for transplantation or research, through written consent and according to Swedish law, were kept in a tissue bank at the corneal donation center at the University Hospital of Umeå, Sweden (Hornhinnebanken Vävnadsinrättningen Laboratoriemedicin Norrlands universitetssjukhus, (http://www.vavnad.se/cms/sites/vavnadsradet/home/hornhinnor/nationellt-arbete-1/umea.html). If these healthy donated corneas were not used for transplantation after their collection, they were delivered to the laboratory for research purpose. The Regional Ethical Review Board in Umeå reviewed the study and determined it to be exempt from the requirement for approval (2010-373-31M). The study was performed according to the principles of the Declaration of Helsinki.

### Isolation and primary culture of human keratocytes

Healthy human corneas were obtained from donated transplantation grafts, as described in the previous section. Samples were scraped using a sterile scalpel to remove any remaining epithelial or endothelial cells before being washed in sterile Hanks’ Balanced Salt Solution (HBSS; Invitrogen, Carlsbad, CA, USA, # 14170–138). Using a scalpel, the cornea was separated into two parts: the central part (round shaped, middle part of the cornea) and the peripheral part (ring shaped part consisting of limbus and surrounding tissue). Each part was then minced with a scalpel and digested in 2 mg/ml collagenase (Clostridopeptidase A, Sigma, St. Louis, MO, USA, # C-1030) diluted in Dulbecco's Modified Eagle Medium: Nutrient Mixture F-12 (DMEM / F-12, Gibco, Carlsbad, CA, USA, # 21331–046) for 30 minutes at 37°C. The samples were then transferred and cultured in DMEM / F-12 medium supplemented with 2% Fetal Bovine Serum (FBS; Gibco, # 16000), 1% penicillin-streptomycin (Gibco, # 15410) and 0.2% L-Glutamine (Gibco, # 25030). The medium was replaced every second to third day until the cells reached confluence. Confluent cells were detached with 0.05% Trypsin-EDTA (Gibco, # 25300) and split in a 1:3 ratio. Cells from passage 3 to 4 were used for experiments. For the experiments, cells were maintained in DMEM / F-12 supplemented with either 2% or 0% FBS (western blots, EIAs, ELISAs, colorimetric and fluorometric assays), or only 2% FBS (immunocytochemistry and real-time qPCR). Central part of the cornea and peripheral part (consisting of limbus + adjacent tissue) were analyzed and compared in this study.

### Human corneal tissue sectioning

Healthy human corneas were fixed in a solution of 4% formaldehyde diluted in 0.1 M Phosphate Buffered Saline (PBS) (pH 7.4) overnight. After three washes in a Tyrode’s solution containing 10% sucrose the sample was mounted on a piece of cardboard with OCT embedding medium (Miles Laboratories, Elkhart, IN, USA) and frozen in liquid nitrogen-chilled propane. The corneas were then cut to 7 μm sections in a cryostat and sections were stored at -24°C until stained.

### Immunocytochemistry

10^4^ keratocytes per well were seeded in 8 well chamber slides (Corning, Corning, NY, USA # 354118) overnight (in DMEM/F12 supplemented with 2% FBS) before being fixed in 2% paraformaldehyde (PFA) diluted in 0.1 M PBS (pH 7.4) for 10 minutes. Cells were permeabilized with 0.25% Triton X-100 in PBS for 10 minutes. Fixed cells were washed repeatedly in PBS and then blocked with 1:20 diluted normal serum (Table 1) corresponding to the host species of the secondary antibody for 15 minutes. After carefully disposing of the serum, cells were incubated with the primary antibody (Table 2) overnight at 4°C. Washing and blocking were repeated and secondary antibody (Table 3) was added for 30 minutes at 37°C. Cells were then washed before being mounted in Vectashield mounting medium for fluorescence (Vector Laboratories, Burlingame, CA, USA # H-1500). A control well was also prepared for each secondary antibody by replacing the primary antibody with PBS. A Zeiss Axioskop 2 plus microscope equipped with epifluorescence and an Olympus DP70 digital camera were used for analysis.

**Table 1:** Normal sera used for immunocytochemistry and immunohistochemistry.

Serum	Code	Source	Dilution
Donkey	017-000-121	Jackson I.R. West Grove, PA, USA	1:20
Rabbit	X0902	Dako, Glostrup, Denmark	1:20
Swine	014-000-121	Jackson I.R. West Grove, PA, USA	1:20

**Table 2:** Primary antibodies used for immunocytochemistry and immunohistochemistry.

Antigen	Code	Source	Type	Dilution
Keratocan	sc-66941	Santa Cruz Biotechnology, Dallas, TX, USA	rabbit	1:50
α-SMA	ab5649	Abcam, Cambridge, UK	rabbit	1:50
Lumican	sc-166871	Santa Cruz Biotechnology, Dallas, TX, USA	mouse	1:50
CD31	M0823	Dako, Glostrup, Denmark	mouse	1:50
SP	8450–0004	Serotec, Oxford, UK	rabbit	1:50
NK-2R	NBP1-00948	Novus Biologicals, Cambridge, UK	rabbit	1:100
NK-1R	sc-5220	Santa Cruz Biotechnology, Dallas, TX, USA	goat	1:100
mAChR M1	sc-9106	Santa Cruz Biotechnology, Dallas, TX, USA	rabbit	1:100
mAChR M2	sc-9107	Santa Cruz Biotechnology, Dallas, TX, USA	rabbit	1:100
mAChR M3	sc-9108	Santa Cruz Biotechnology, Dallas, TX, USA	rabbit	1:100
mAChR M4	sc-9109	Santa Cruz Biotechnology, Dallas, TX, USA	rabbit	1:100
mAChR M5	sc-9110	Santa Cruz Biotechnology, Dallas, TX, USA	rabbit	1:100
TH	sc-14007	Santa Cruz Biotechnology, Dallas, TX, USA	rabbit	1:50
Glutamate	G6642	Sigma, St. Louis, MO, USA	rabbit	1:100
NKA	H-046-15	Phoenix Pharmaceuticals, Burlingame, CA, USA	rabbit	1:100
ChAT	AB143	Millipore, Billerica, MA, USA	rabbit	1:100
NMDAR1	ab134308	Abcam, Cambridge, UK	mouse	1:250
α1 adrenergic receptor (ADRA1)	ab3462	Abcam, Cambridge, UK	rabbit	1:500
β2 adrenergic receptor (ADRB2)	ab61778	Abcam, Cambridge, UK	rabbit	1:250
Dopamine D2 Receptor (DRD2)	ab32349	Abcam, Cambridge, UK	goat	1:250
CD45	ab10558	Abcam, Cambridge, UK	rabbit	1:500

**Table 3:** Secondary antibodies used for immunocytochemistry and immunohistochemistry.

Secondary antibody	Code	Source	Dilution
Alexa Fluor 488, donkey anti-goat	A-11055	Invitrogen, Carlsbad, CA, USA	1:300
TRITC-conjugated rabbit anti-mouse	R0270	Dako, Glostrup, Denmark	1:20
TRITC-conjugated swine anti-rabbit	R0156	Dako, Glostrup, Denmark	1:40

### Immunohistochemistry

Tissue sections were fixed in 2% PFA and permeabilized with 1% Triton X-100 for 20 minutes. Slides were then washed 3 times in PBS and blocked with 1:20 diluted normal serum (Table 1) for 15 minutes. After carefully disposing of the serum, cells were incubated with the primary antibody (Table 2) overnight at 4°C. The same antibodies were used as for immunocytochemistry. Washing and blocking were repeated and secondary antibody (Table 3) was added for 30 minutes at 37°C. Sections were then washed before being mounted in Vectashield mounting medium for fluorescence (Vector Laboratories, # H-1500). A control slide was also prepared for each secondary antibody by replacing the primary antibody with PBS. A Zeiss Axioskop 2 plus microscope equipped with epifluorescence and an Olympus DP70 digital camera were used for analysis.

### Measurements of neuropeptides and neurotransmitters

250,000 keratocytes were seeded into 6 wells plates in triplicates in DMEM / F-12 medium supplemented with either 2% or 0% FBS. After 24 h of culture, supernatants were collected and cells were lysed in RIPA lysis buffer. Neuropeptides and neurotransmitters were measured using following kits according to manufacturer’s specifications: Substance P EIA kit (Phoenix Pharmaceuticals, Burlingame, CA, USA, # EK-061-05), Neurokinin A EIA kit (RayBiotech, Norcross, GA, USA, # EIA-NEA1), Amplex Acetylcholine/Acetlycholinesterase Assay Kit (Life Technologies, Carlsbad, CA, USA), Glutamate Assay kit (Abcam, # 83389), and Adrenaline/Noradrenaline/Dopamine 3-CAT ELISA (Labor Diagnostika Nord, Nordhorn, Germany, # BA E-5600).

### Western blot

250,000 keratocytes were seeded into 6 well plates in DMEM / F-12 medium supplemented with either 2% or 0% FBS. After 24h of culture, cells were washed with PBS and frozen at -80°C overnight. Cells were lysed in RIPA lysis buffer supplemented with 0.5% Proteinase inhibitor cocktail (Sigma, # P1860) and diluted in Laemmli Sample buffer supplemented with 2-mercaptoethanol. The cell lysates were separated by sodium dodecyl sulfate / polyacrylamide gel electrophoresis and transferred to PVDF membranes. Blots were incubated with primary antibodies listed in Table 4. The antigens were detected with horseradish peroxidase-conjugated secondary antibodies listed in Table 5. The images were taken by Odyssey Fc imaging system (LI-COR, Lincoln, NE, USA). Densitometry was performed using Image J analysis software (NIH).

**Table 4:** Primary antibodies used in western blot analysis.

Antigen	Code	Source	Type	Dilution
Keratocan	sc-66941	Santa Cruz Biotechnology, Dallas, TX, USA	rabbit	1:200
Lumican	sc-166871	Santa Cruz Biotechnology, Dallas, TX, USA	mouse	1:200
CD34	sc-9095	Santa Cruz Biotechnology, Dallas, TX, USA	rabbit	1:200
ALDH	ab52492	Abcam, Cambridge, UK	rabbit	1:500
Procollagen I (Pro-COL1A1)	sc-8782	Santa Cruz Biotechnology, Dallas, TX, USA	goat	1:200
Collagen I (COL1A1)	ab34710	Abcam, Cambridge, UK	rabbit	1:500
NK-1R	MAB6687	R&D Systems, Abingdon, UK	mouse	1:100
NK-2R	NBP1-00948	Novus Biologicals, Cambridge, UK	rabbit	1:100
TH	sc-14007	Santa Cruz Biotechnology, Dallas, TX, USA	rabbit	1:200
Dopamine D2 Receptor (DRD2)	ab32349	Abcam, Cambridge, UK	goat	1:500
mAChR M1	sc-9106	Santa Cruz Biotechnology, Dallas, TX, USA	rabbit	1:200
mAChR M3	sc-9108	Santa Cruz Biotechnology, Dallas, TX, USA	rabbit	1:200
mAChR M4	sc-9109	Santa Cruz Biotechnology, Dallas, TX, USA	rabbit	1:200
mAChR M5	sc-9110	Santa Cruz Biotechnology, Dallas, TX, USA	rabbit	1:200
NMDAR1	ab134308	Abcam, Cambridge, UK	mouse	1:500

**Table 5:** HRP-linked secondary antibodies.

Secondary antibody	Code	Source	Dilution
Anti-rabbit IgG HRP-linked	7074	Cell Signaling, Danvers, MA, USA	1:2000
Anti-mouse IgG HRP-linked	7076	Cell Signaling, Danvers, MA, USA	1:2000
Anti-goat IgG HRP-linked	sc-2020	Santa Cruz Biotechnology, Dallas, TX, USA	1:2000

### Real-time qPCR

250,000 keratocytes were seeded into 6 wells plates in triplicates in DMEM/F12 supplemented with 2% FBS. After 24 h RNA was extracted using RNeasy Mini Kit (Qiagen, Venlo, The Netherlands, #74104). 900 ng of RNA was reverse transcribed into cDNA using High Capacity cDNA Reverse Transcription Kit (Life Technologies, Carlsbad, CA, USA, # 4368814). To determine the gene expression TaqMan Gene Expression Assays (Applied Biosystems, Carlsbad, USA) were used. cDNA obtained from 40 ng of RNA was run in duplicates by ViiA 7 Real-Time PCR system (Applied Biosystems), and analyzed by ViiA 7 Software (Applied Biosystems). Expression of genes of interest was normalized to the expression of 18S housekeeping gene. Analyzed genes are listed in Table 6.

**Table 6:** Gene expression assays.

Gene	ID
ADRA1B	Hs00171263_m1
ADRB2	Hs00240532_s1
CHRM1	Hs00265195_s1
CHRM2	Hs00265208_s1
CHRM3	Hs00265216_s1
CHRM4	Hs00265219_s1
CHRM5	Hs00255278_s1
DDC	Hs01105048_m1
DRD2	Hs00241436_m1
GLS	Hs00248163_m1
GOT	Hs00157798_m1
TAC1	Hs00243225_m1
TACR1	Hs00185530_m1
TACR2	Hs00169052_m1

### Statistical analysis

Data represent mean ± SD of three replicates. Independent samples *t*-test or one-way ANOVA with Bonferroni post hoc test were applied to determine if there was a statistically significant difference between samples. GraphPad Prism 5 software was used for data analysis. Significance was predetermined at p<0.05. Experiments were performed successfully at least 3 times. Primary cultures of keratocytes obtained from different cornea donors were assessed individually.

## Results

### Characterization of isolated keratocytes *in vitro*

**Fig 1:**
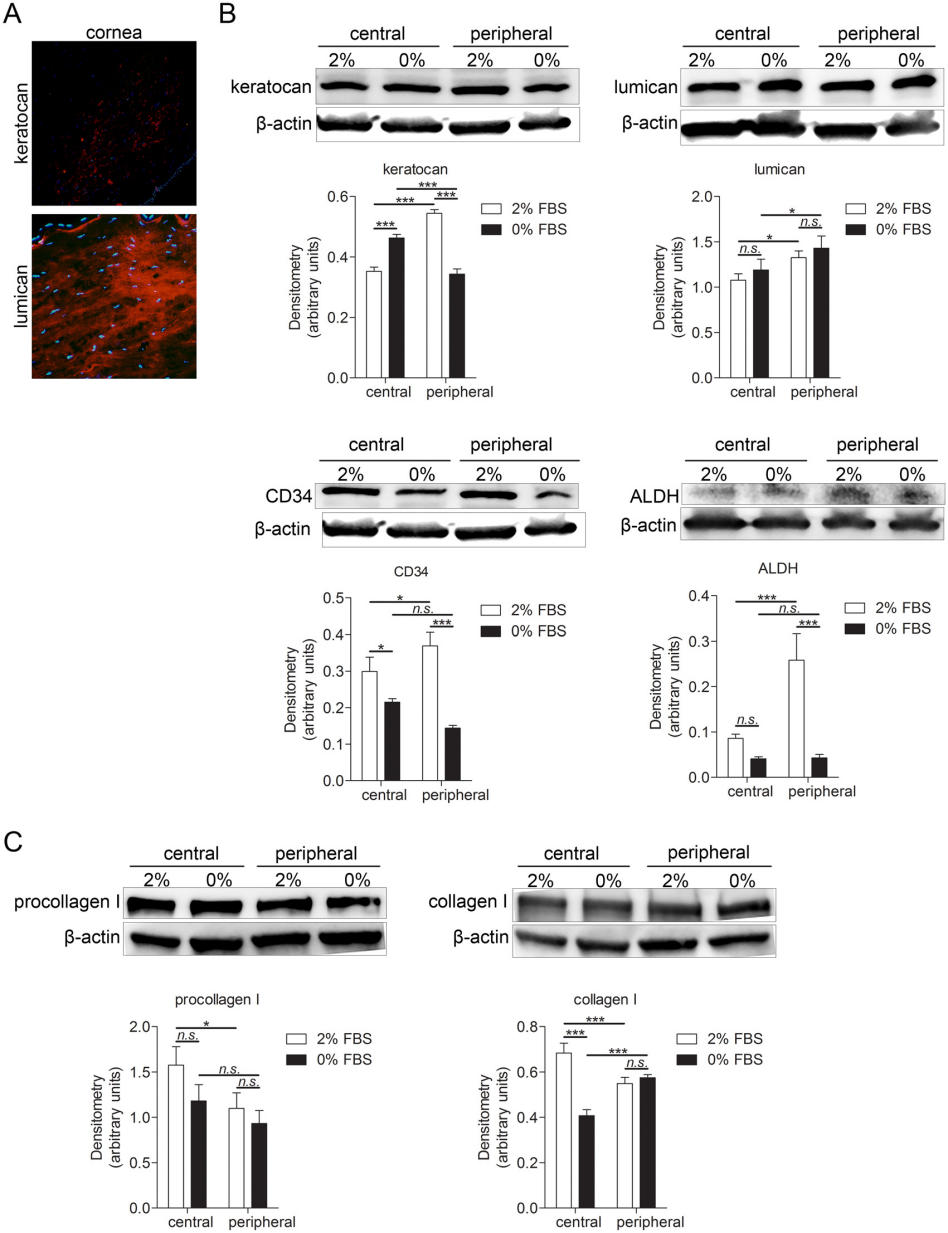
Characterization of isolated keratocytes and keratocytes in cornea tissue sections. **(A)** Human corneal sections were characterized by immunohistochemistry. Sections were double stained for the nuclei (DAPI; blue) together with keratocan (top panel) or lumican (bottom panel) (both TRITC; red). Both keratocan and lumican were expressed by keratocytes in corneal sections. **(B-C)** 250,000 cells were grown for 24h in 6 well plates in DMEM/F12 supplemented with either 2% or 0% FBS and subjected to western blot analysis. **(B)** Four keratocyte markers were analyzed: keratocan (50 kDa), lumican (46 kDa), CD34 (90–120 kDa) and ALDH (55 kDa). Keratocan was expressed in cultured cells. Its expression was significantly higher in peripheral keratocytes cultured in 2% FBS than in central keratocytes grown in 2% FBS (***p<0.001). Central keratocytes grown in 0% FBS expressed more keratocan than central keratocytes grown in 2% FBS (***p<0.001) and peripheral keratocytes grown in 0% FBS (***p<0.001). However, peripheral keratocytes grown in 0% FBS had significantly lower expression of keratocan than peripheral keratocytes grown in 2% FBS (***p<0.001), Lumican was expressed in cultured cells. Peripheral keratocytes expressed significantly more lumican than central keratocytes (*p<0.05 for 2% FBS, *p<0.05 for 0% FBS). CD34 was expressed in cultured keratocytes. Its expression was significantly higher in peripheral keratocytes grown in 2% FBS than in central keratocytes grown in 2% FBS (*p<0.05) and peripheral keratocytes grown in 0% FBS (***p<0.001). CD34 expression was also significantly higher in central keratocytes grown in 2% FBS than in central keratocytes grown in 0% FBS (*p<0.05). ALDH was expressed in cultured keratocytes but at low levels. Peripheral keratocytes grown in 2% FBS expressed significantly more ALDH than central keratocytes grown in 2% FBS (***p<0.001) and peripheral keratocytes grown in 0% FBS (***p<0.001). (C) Expression of procollagen I (140–210 kDa) and collagen I (139 kDa) was analyzed in cultured keratocytes. Procollagen I was expressed by cultured keratocytes. Its expression was significantly higher in central keratocytes grown in 2% FBS than in peripheral keratocytes grown in same conditions (*p<0.05). Collagen I expression was significantly higher in central keratocytes grown in 2% FBS than in central keratocytes grown in 0% FBS (***p<0.001) and peripheral keratocytes grown in 2% FBS (***p<0.001). Also, collagen I expression was significantly lower in central keratocytes grown in 0% FBS than in peripheral keratocytes grown in same conditions (***p<0.001). β-actin (42 kDa) served as a loading control. Values are means± SD.

Immunohistochemistry of the human corneal tissue showed that the cornea expresses keratocan, a cornea-specific keratan sulfate proteoglycan, [31], and lumican which is a keratan sulfate proteoglycan produced by keratocytes [31](Fig 1A). In order to determine the characteristics and phenotype of cultured corneal cells, and how the culturing conditions affect the cultured cells phenotype, four keratocyte markers were used: keratocan, lumican, CD34 (hematopoietic stem cell marker and an adhesion molecule [32] and aldehyde dehydrogenase (ALDH; corneal crystalline which contributes to maintaining corneal transparency [33]). Western blot and densitometry analyses were performed. Two culture conditions were tested. Central and peripheral keratocytes were cultured in either DMEM/F12 medium supplemented with 2% FBS or 0% FBS. Central keratocytes grown in these two conditions, as well as peripheral keratocytes grown in these two conditions, were compared. Moreover, comparison was made between central and peripheral keratocytes. Keratocan was expressed abundantly in cultured cells. Its expression was significantly higher in peripheral keratocytes cultured in 2% FBS than in central keratocytes grown in 2% FBS. Central keratocytes grown in 0% FBS expressed more keratocan than central keratocytes grown in 2% FBS and peripheral keratocytes grown in 0% FBS. However, peripheral keratocytes grown in 0% FBS had significantly lower expression of keratocan when compared to peripheral keratocytes grown in 2% FBS. Lumican was also expressed abundantly in cultured cells. Peripheral keratocytes expressed significantly more lumican than central keratocytes. CD34 was also expressed in cultured keratocytes. Its expression was significantly higher in peripheral keratocytes grown in 2% FBS than in central keratocytes grown in 2% FBS and peripheral keratocytes grown in 0% FBS. CD34 expression was also significantly higher in central keratocytes grown in 2% FBS in comparison to central keratocytes grown in 0% FBS. ALDH was expressed in cultured keratocytes but at very low levels. Peripheral keratocytes grown in 2% FBS expressed significantly more ALDH than central keratocytes grown in 2% FBS and peripheral keratocytes grown in 0% FBS (Fig 1B). Moreover, expression of procollagen I and collagen I was assessed. Procollagen I was significantly higher in central keratocytes grown in 2% FBS than in peripheral keratocytes grown in same conditions. Collagen I expression was significantly higher in central keratocytes grown in 2% FBS than in central keratocytes grown in 0% FBS and peripheral keratocytes grown in 2% FBS. Also, collagen I expression was significantly lower in central keratocytes grown in 0% FBS than in peripheral keratocytes grown in same conditions (Fig 1C). Additionally, cultured keratocytes did not express CD45, a common marker for bone marrow-derived cells, CD31, a marker of endothelial cells and expressed only small amount of α-SMA, a marker of myofibroblasts (data not shown).

### Human keratocytes express genes coding for neuropeptides, and for catecholamine and glutamate synthesizing enzymes

In order to study if cultured keratocytes express genes coding for neuropeptides or for enzymes taking part in the synthesis of catecholamines and glutamate, qPCR was performed (S1 Fig). The gene coding for substance P and neurokinin A (TAC1) was present both in central and peripheral keratocytes. The DDC gene, which codes for DOPA decarboxylase, an enzyme involved in catecholamine synthesis, was also present in both central and peripheral keratocytes. The glutaminase gene (GLS), which codes for glutaminase that generates glutamate from glutamine, and the GOT gene coding for aspartate aminotransferase, which plays a role in amino acid metabolism, were both present in central and peripheral keratocytes.

### Human keratocytes express genes coding for receptors of neuropeptides, catecholamines, and acetylcholine

Genes coding for receptors of neuropeptides, catecholamines, and ACh were analyzed by qPCR (S1 Fig). TACR1 gene, coding for the preferred receptor of SP, the NK-1R, and TACR2 gene, which is the gene for the preferred receptor of neurokinin A (NK-2R), were present in both central and peripheral keratocytes. mRNA for adrenaline and noradrenaline adrenoreceptors α_1_ (ADRA1B) and β_2_ (ADRB2) were present in central keratocytes and peripheral keratocytes. DRD2 gene, coding for another catecholamine receptor, the dopamine receptor D_2_, was also present in both central and peripheral keratocytes. Genes for muscarinic acetylcholine receptors–CHRM1 (for mAChR M_1_), CHRM2 (for mAChR M_2_), CHRM3 (for mAChR M_3_), CHRM4 (for mAChR M_4_), and CHRM5 (for mAChR M_5_) were found in central and peripheral keratocytes.

### Human keratocytes produce substance P and neurokinin A, and express their preferred receptors

**Fig 2:**
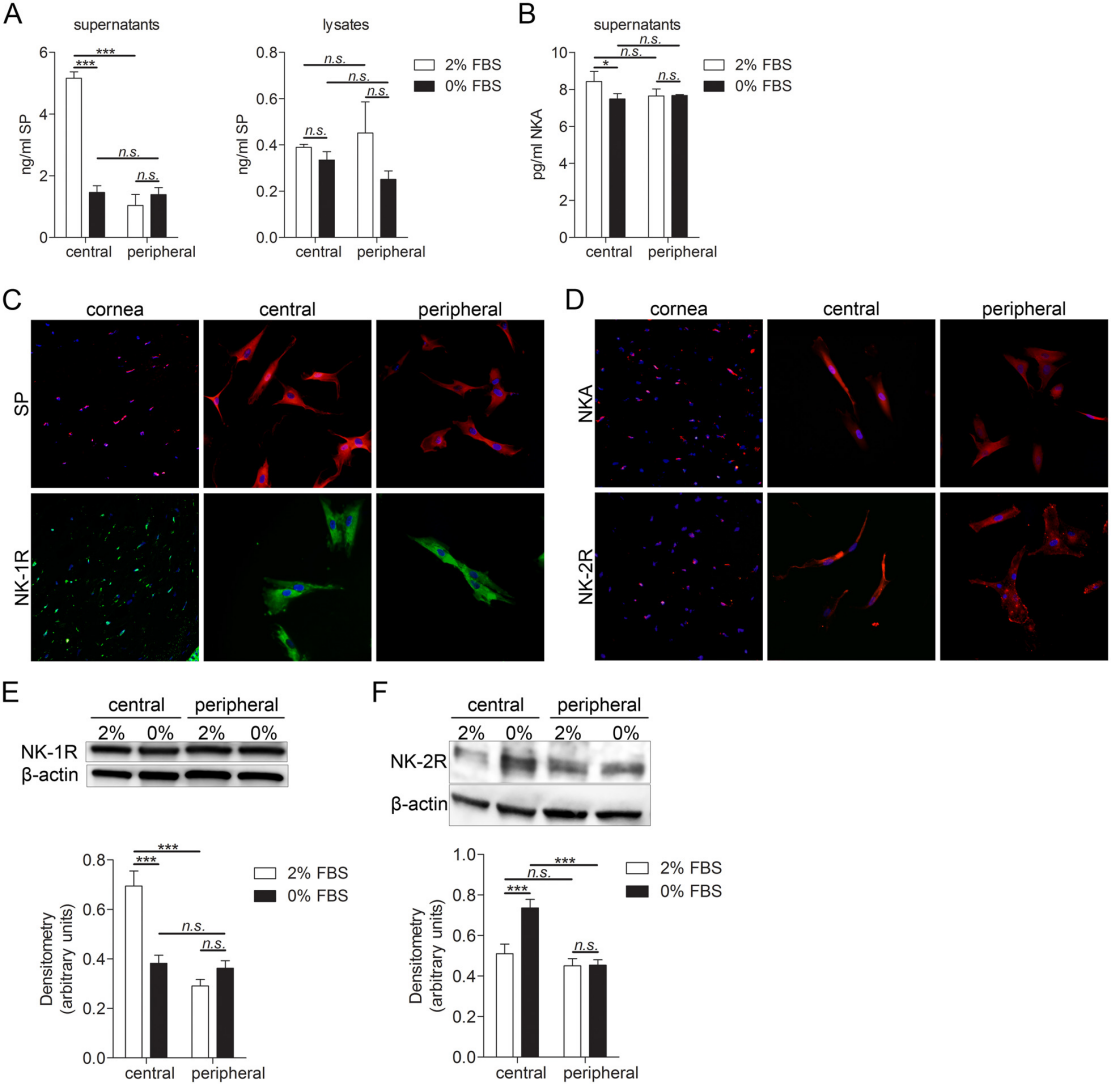
Substance P and neurokinin A expression in keratocytes of cornea tissue sections and in cultured primary keratocytes. **(A-B)** Substance P **(A)** and neurokinin A **(B)** EIA of central and peripheral cultured keratocytes. 250,000 cells were grown for 24h in 6 well plates in DMEM/F12 supplemented with either 2% or 0% FBS. Supernatant was collected, cells were lysed and subjected to EIA. **(A)** SP levels were significantly higher in supernatants of central keratocytes grown in medium supplemented with 2% FBS than in peripheral keratocytes grown in medium supplemented with 2% FBS (***p<0.001). SP secretion was significantly higher in central keratocytes grown in 2% FBS than central keratocytes grown in 0% FBS (***p<0.001). There were no significant differences in SP levels in lysates of keratocytes. **(B)** NKA secretion was significantly higher in central keratocytes grown in 2% FBS than in central keratocytes grown in 0% FBS (*p<0.05). **(C)** Keratocytes in sections (left panel), as well as cultured central (middle panel) and peripheral (right panel) keratocytes, were labeled with an antibody targeting SP (top row; TRITC; red) and double stained with DAPI to visualize the nuclei (blue). Sections and cultured cells were also labeled with an antibody targeting full-length NK-1R (bottom row; Alexa Fluor 488; green) and double stained with DAPI. Both the keratocytes in tissue sections and the cultured keratocytes expressed SP and a full-length version of NK-1R. **(D)** Keratocytes in sections (left panel), as well as cultured central (middle panel) and peripheral (right panel) keratocytes, were labeled with an antibody targeting NKA (top row; TRITC; red) and NK-2R (bottom row; TRITC; red) and double stained with DAPI. Both the keratocytes in tissue sections and the cultured keratocytes expressed NKA and NK-2R. **(E-F)** 250,000 central keratocytes or peripheral keratocytes grown in DMEM/F12 supplemented with either 2% or 0% FBS were subjected to western blot analysis for NK-1R (46 kDa) and NK-2R (48 kDa) expression. **(E)** Expression of NK-1R was significantly higher in central keratocytes grown in 2% FBS than in peripheral keratocytes grown in same conditions (***p<0.001). Expression of NK-1R was also significantly higher in central keratocytes grown in 2% FBS than in central keratocytes grown in 0% FBS (***p<0.001). **(F)** Expression of NK-2R was significantly higher in central keratocytes grown in 0% FBS than in peripheral keratocytes grown in same conditions (***p<0.001). NK-2R expression was significantly higher in central keratocytes grown in 0% FBS than in central keratocytes grown in 2% (***p<0.001). n.s. (non significant; p>0.05). β-actin (42 kDa) served as a loading control. Values are means ± SD.

Presence and differences in amounts of the neuropeptides SP and NKA in cultures of human keratocytes was measured by EIA. Two culture conditions were tested. Central and peripheral keratocytes were cultured in either DMEM/F12 medium supplemented with 2% FBS or 0% FBS. Central keratocytes grown in these two conditions, as well as peripheral keratocytes grown in these two conditions, were compared. Moreover, comparison was made between central and peripheral keratocytes. SP levels were significantly higher in supernatants of central keratocytes grown in medium supplemented with 2% FBS than in peripheral keratocytes grown in medium supplemented with 2% FBS. SP secretion was significantly higher in central keratocytes grown in 2% FBS than central keratocytes grown in 0% FBS. However, there were no significant differences in SP levels in lysates of keratocytes (Fig 2A). NKA secretion was also significantly higher in central keratocytes grown in 2% FBS when compared to central keratocytes grown in 0% FBS (Fig 2B). Expression of SP and of its preferred receptor, NK-1R, was furthermore analyzed by immunohistochemistry and immunocytochemistry. Both the keratocytes in tissue sections and the cultured keratocytes expressed SP and a full-length version of NK-1R, composed of 407 amino acid residues (Fig 2C). Expression of NKA and of its receptor, NK-2R, was also analyzed by immunohistochemistry and immunocytochemistry. Both the keratocytes in tissue sections and the cultured keratocytes expressed NKA and NK-2R (Fig 2D). To quantify differences in expression of NK-1R and NK-2R between the different culturing conditions and central and peripheral keratocytes, western blot and densitometry analyses were performed. Expression of NK-1R was significantly higher in central keratocytes grown in 2% FBS when compared to peripheral keratocytes grown in same conditions. Expression of NK-1R was also significantly higher in central keratocytes grown in 2% FBS than in central keratocytes grown in 0% FBS (Fig 2E). On the other hand, expression of NK-2R was significantly higher in central keratocytes grown in 0% FBS than in peripheral keratocytes grown in same conditions. NK-2R expression was significantly higher in central keratocytes grown in 0% FBS than in central keratocytes grown in 2% (Fig 2F).

### Human keratocytes produce catecholamines and express their receptors

**Fig 3:**
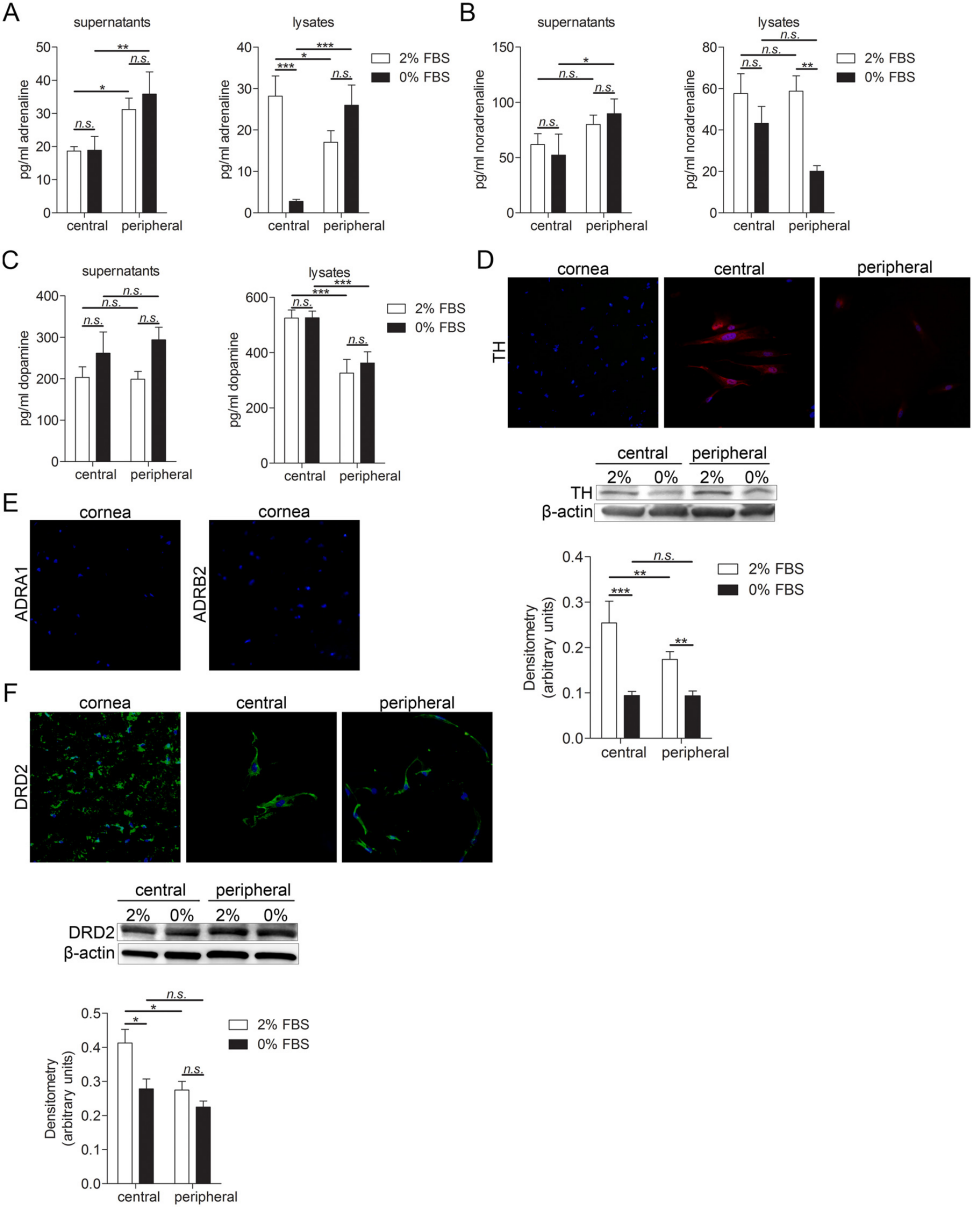
Catecholamines and their receptors expression in keratocytes of cornea tissue sections and cultured primary keratocytes. (**A-C**) Adrenaline, noradrenaline and dopamine ELISAs of lysates and supernatants of central and peripheral cultured keratocytes. 250,000 cells were grown for 24h in 6 well plates in DMEM/F12 supplemented with either 2% or 0% FBS. Supernatant was collected, cells were lysed and subjected to ELISA. **(A)** Levels of secreted adrenaline were significantly higher in peripheral keratocytes grown in both 2% and 0% FBS than in central keratocytes grown in same conditions (*p<0.05 for 2% FBS and **p<0.01 for 0% FBS). Levels of intracellular adrenaline were significantly higher in central keratocytes grown in 2% FBS than in peripheral keratocytes grown in 2% FBS (*p<0.05). Central keratocytes grown in 2% FBS had higher levels of adrenaline than central keratocytes grown in 0% FBS (***p<0.001). Moreover, peripheral keratocytes grown in 0% FBS had higher levels of adrenaline than central keratocytes grown in same the same condition (***p<0.001). **(B)** Noradrenaline was secreted from both central and peripheral keratocytes and was also present in cell lysates. Secreted noradrenaline levels were significantly higher in peripheral keratocytes grown in 0% FBS than in central keratocytes grown in same conditions (*p<0.05). Intracellular levels of noradrenaline were significantly higher in peripheral keratocytes grown in 2% FBS than peripheral keratocytes grown in 0% FBS. **(C)** Dopamine was secreted from both central and peripheral keratocytes. Dopamine levels were significantly higher in lysates of central keratocytes than peripheral keratocytes (***p<0.001 for both 2% FBS and 0% FBS). **(D)** Keratocytes in sections (left panel), as well as cultured central (middle panel) and peripheral (right panel) keratocytes, were labeled with an antibody targeting tyrosine hydroxylase (TH; TRITC; red) and double stained with DAPI to visualize the nuclei (blue). Cultured keratocytes expressed TH whereas keratocytes in tissue sections did not. 250,000 central or peripheral keratocytes grown in either 2% FBS or 0% FBS were lysed and expression of TH (60 kDa) was analyzed by western blot. Both central and peripheral keratocytes grown in 2% FBS expressed more TH than cells grown in 0% FBS (***p<0.001 for central keratocytes, **p<0.01 for peripheral keratocytes). Moreover, central keratocytes grown in 2% FBS expressed more TH than peripheral keratocytes grown in same conditions (**p<0.01). β-actin (42 kDa) served as a loading control. **(E)** Sections were labeled with adrenergic receptor antibodies, ADRA1 (left; TRITC, red) and ADRB2 (right; TRITC, red) and double stained with DAPI (blue). Keratocytes in tissue sections did not express either of the adrenergic receptors. **(F)** Keratocytes in sections (left panel), as well as cultured central (middle panel) and peripheral (right panel) keratocytes, were labeled with an antibody targeting dopamine receptor DRD2 (Alexa Fluor 488; green) and double stained with DAPI to visualize the nuclei (blue). DRD2 was expressed in both keratocytes in tissue sections and in cultured keratocytes. 250,000 central or peripheral keratocytes grown in either 2% FBS or 0% FBS were lysed and expression of DRD2 (51 kDa) was analyzed by western blot. Central keratocytes grown in 2% FBS had significantly higher expression of DRD2 than both central keratocytes grown in 0% FBS (*p<0.05) and peripheral cells grown in 2% FBS (*p<0.05). β-actin (42 kDa) served as a loading control. Values are means ± SD.

Presence and differences in amounts of catecholamines (adrenaline, noradrenaline, and dopamine) was measured by ELISA. Again, two culture conditions were tested. Central and peripheral keratocytes were cultured in either DMEM/F12 medium supplemented with 2% FBS or 0% FBS. Central keratocytes grown in these two conditions, as well as peripheral keratocytes grown in these two conditions, were compared. Moreover, comparison was made between central and peripheral keratocytes. Adrenaline was present in both culture supernatant and lysates of cultured central and peripheral keratocytes, with significantly higher levels in supernatants collected from peripheral cells. However, in cell lysates levels of intracellular adrenaline were significantly higher in central keratocytes grown in 2% FBS in comparison with peripheral keratocytes grown in 2% FBS. Central keratocytes grown in 2% FBS showed higher levels of adrenaline than central keratocytes grown in 0% FBS. Moreover, peripheral keratocytes grown in 0% FBS had higher levels of adrenaline than central keratocytes grown in same conditions (Fig 3A). Noradrenaline was secreted from both central and peripheral keratocytes, and was also present in cell lysates. Peripheral keratocytes grown in 0% FBS secreted significantly higher amounts of noradrenaline than central keratocytes grown in same conditions. Intracellular levels of noradrenaline were significantly higher in peripheral keratocytes grown in 2% FBS than in peripheral keratocytes grown in 0% FBS (Fig 3B). Dopamine was secreted from both central and peripheral keratocytes and its levels were significantly higher in lysates of central keratocytes than peripheral keratocytes. Expression of tyrosine hydroxylase (TH), the enzyme responsible for catalyzing the conversion of the amino acid L-tyrosine to L-DOPA (precursor of adrenaline, noradrenaline, and dopamine), was analyzed by immunohistochemistry and immunocytochemistry. The cultured keratocytes expressed TH whereas keratocytes in tissue sections did not. In order to quantify and compare TH expression under different culturing conditions and two types of keratocytes (central vs. peripheral), western blot and densitometry analyses were performed. Both central and peripheral keratocytes grown in 2% FBS expressed more TH than cells grown in 0%. Moreover, central keratocytes grown in 2% FBS expressed more TH than peripheral keratocytes grown in same conditions (Fig 3D). Adrenaline and noradrenaline adrenoreceptors α_1_ and β_2_ (ADRA1, ADRB2, respectively) were not expressed in corneal tissue sections (Fig 3E) Expression of these receptors in cultured keratocytes was inconclusive. However, dopamine D2 receptor (DRD2) was expressed in both keratocytes in tissue sections and in cultured keratocytes. Western blot and densitometry analyses showed that central keratocytes grown in 2% FBS had significantly higher expression of DRD2 than both central keratocytes grown in 0% FBS and peripheral cells grown in 2% FBS (Fig 3F). The expression of TH in cultured cells but not in cells of tissue sections might be a result of culturing conditions.

### Human keratocytes produce acetylcholine and express muscarinic acetylcholine receptors

**Fig 4:**
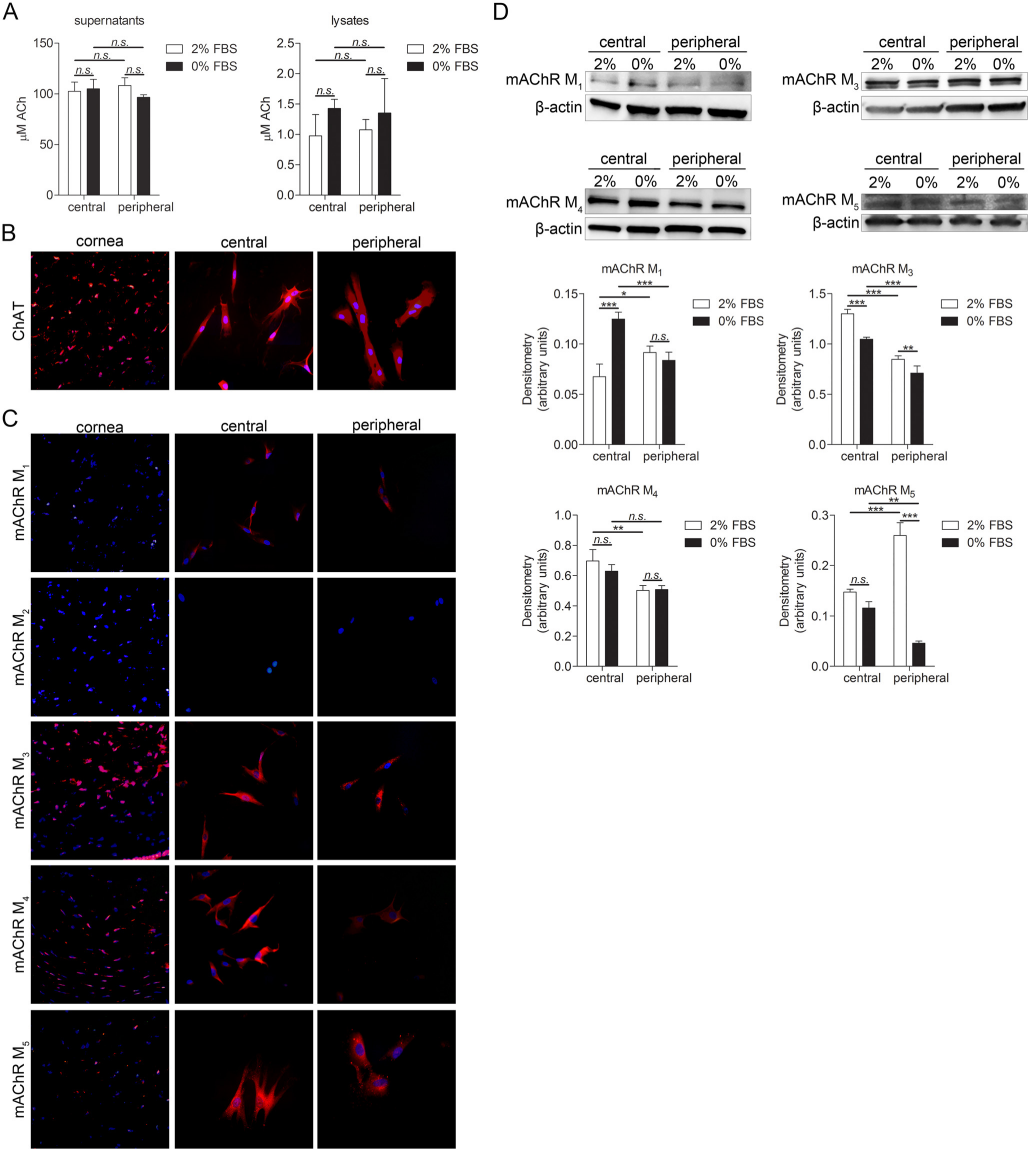
Acetylcholine and its receptors expression in keratocytes. **(A)** 250,000 cells were grown for 24h in 6 well plates in DMEM/F12 supplemented with either 2% or 0% FBS. Supernatant was collected and subjected to acetylcholine (ACh) assay and cells were lysed. Both central and peripheral keratocytes secreted ACh (*n*.*s*.; p≥0.05). ACh was also present in both central and peripheral keratocyte lysates (*n*.*s*.; p≥0.05). **(B)** Keratocytes in sections (left panel), as well as cultured central (middle panel) and peripheral (right panel) keratocytes, were labeled with an antibody targeting choline acetyltransferase (ChAT; TRITC; red), and double stained with DAPI to visualize the nuclei (blue). Both the keratocytes in tissue sections and the cultured keratocytes expressed ChAT. **(C)** Keratocytes in sections (left panel), as well as cultured central (middle panel) and peripheral (right panel) keratocytes, were labeled with muscarinic acetylcholine receptors (mAChR M_1_, mAChR M_2_, mAChR M_3_, mAChR M_4_, and mAChR_5_; TRITC; red) and double stained with DAPI (blue). mAChR M_1_ was present in cultured cells but not in tissue sections, mAChR M_2_ was absent in both cultured cells and tissue sections. Remaining receptors (mAChR M_3_, mAChR M_4_, and mAChR M_5_) were expressed in both tissue sections and cultured cells. **(D)** 250,000 central or peripheral keratocytes grown in either 2% FBS or 0% FBS were lysed and expression of muscarinic acetylcholine receptors (mAChR M_1_ [52 kDa], mAChR M_3_ [75 kDa], mAChR M_4_ [74 kDa], and mAChR_5_ [60 kDa]_;_ mAChR M_2_ was not expressed) was analyzed by western blot. mAChR M_1_ expression was significantly higher in central keratocytes grown in 0% FBS than in central keratocytes grown in 2% FBS (***p<0.001), significantly higher in peripheral keratocytes grown in 2% FBS than in central keratocytes grown in same the condition (*p<0.05), and significantly higher in central keratocytes grown in 0% FBS than in peripheral keratocytes grown in the same condition (***p<0.001). mAChR M_3_ expression was significantly higher in central keratocytes grown in 2% FBS than in both central keratocytes grown in 0% FBS (***p<0.001) and peripheral keratocytes grown in 2% FBS (***p<0.001). Central keratocytes grown in 0% FBS expressed significantly more mAChR M_3_ than peripheral keratocytes grown in same conditions (***p<0.001) and peripheral keratocytes grown in 2% FBS expressed more mAChR M_3_ than peripheral cells grown in 0% FBS (**p<0.01). Expression of mAChR M_4_ was significantly higher in central keratocytes grown in 2% FBS than in peripheral keratocytes grown in same conditions (**p<0.01). Expression of mAChR_5_ was significantly higher in peripheral keratocytes grown in 2% FBS than in both central keratocytes grown in same conditions (***p<0.001) and peripheral keratocytes grown in 0% FBS (***p<0.001). mAChR_5_ expression was also significantly higher in central keratocytes grown in 0% FBS than in peripheral keratocytes grown in same conditions (**p<0.01). β-actin (42 kDa) served as a loading control. Values are means ± SD.

Presence and differences in amount of ACh was measured by fluorometric assay. Two culture conditions were tested. Central and peripheral keratocytes were cultured in either DMEM/F12 medium supplemented with 2% FBS or 0% FBS. Central keratocytes grown in these two conditions, as well as peripheral keratocytes grown in these two conditions, were compared. Moreover, comparison was made between central and peripheral keratocytes. Both central and peripheral keratocytes secreted ACh, and ACh was also present in both central and peripheral keratocyte lysates (Fig 4A). Expression of choline acetyltransferase (ChAT)–an enzyme that is crucial for ACh synthesis–was analyzed by immunohistochemistry and immunocytochemistry. Both the keratocytes in tissue sections and the cultured keratocytes expressed ChAT (Fig 4B). Muscarinic acetylcholine receptors: mAChR M_1_, mAChR M_2_, mAChR M_3_, mAChR M_4_, and mAChR M_5_ expression was analyzed by immunohistochemistry and immunocytochemistry (Fig 4C). mAChR M_1_ was present in cultured cells but not in tissue sections, mAChR M_2_ was absent in both cultured cells and tissue sections. The remaining receptor subtypes (mAChR M_3_, mAChR M_4_, and mAChR M_5_) were expressed in both tissue sections and cultured cells. Presence of mAChR M_1_ in culture but its absence in tissue sections might, again, be a result of culturing conditions and/or stressed cells. In order to quantify and compare expression levels of muscarinic acetylcholine receptors under different culturing conditions and two types of keratocytes, western blot and densitometry analyses were performed. mAChR M_1_ expression was significantly higher in central keratocytes grown in 0% FBS than in central keratocytes grown in 2% FBS, and also significantly higher in peripheral keratocytes grown in 2% FBS than in central keratocytes grown in same conditions, and finally significantly higher in central keratocytes grown in 0% FBS than in peripheral keratocytes grown in same condition. mAChR M_3_ expression was significantly higher in central keratocytes grown in 2% FBS than in both central keratocytes grown in 0% FBS and peripheral keratocytes grown in 2% FBS. Central keratocytes grown in 0% FBS expressed significantly more mAChR M_3_ than peripheral keratocytes grown in same the condition, and peripheral keratocytes grown in 2% FBS expressed more mAChR M_3_ than peripheral cells grown in 0% FBS. Expression of mAChR M_4_ was significantly higher in central keratocytes grown in 2% FBS than in peripheral keratocytes grown in same the condition. Expression of mAChR_5_ was significantly higher in peripheral keratocytes grown in 2% FBS than in both central keratocytes grown in the same condition and in peripheral keratocytes grown in 0% FBS. mAChR_5_ expression was also significantly higher in central keratocytes grown in 0% FBS than in peripheral keratocytes grown in same the condition (Fig 4D).

### Human keratocytes produce glutamate and express glutamate receptor NMDAR1

**Fig 5:**
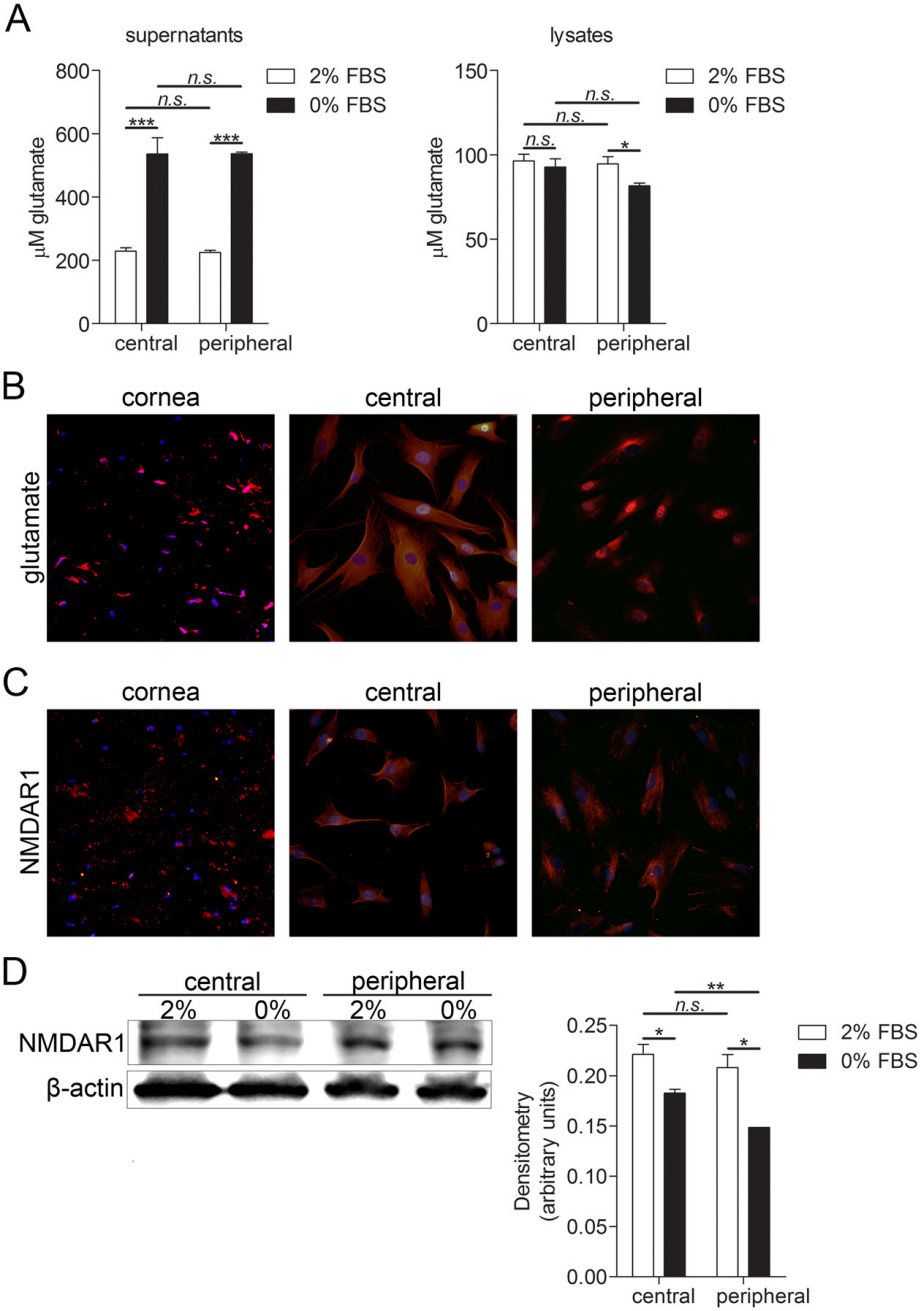
Glutamate and NMDAR1 expression in keratocytes. **(A)** Glutamate assay of cultured central and peripheral keratocytes. 250,000 cells were grown for 24h in 6 well plates in DMEM/F12 supplemented with either 2% or 0% FBS. Supernatant was collected, cell were lysed and subjected to the glutamate assay. Levels of glutamate were significantly higher in central and peripheral keratocyte culture supernatants collected from cells grown in 0% FBS than in supernatants collected from cells grown in 2% (***p<0.001 for central and ***p<0.001 for peripheral keratocytes). Levels of glutamate in cell lysates were significantly higher in peripheral keratocytes grown in 2% FBS than in peripheral keratocytes grown in 0% FBS (*p<0.05) **(B)** Keratocytes in sections (left panel), as well as cultured central (middle panel) and peripheral (right panel) keratocytes, were labeled with glutamate antibody (TRITC; red) and double stained with DAPI to visualize the nuclei (blue). Glutamate was expressed in both keratocytes in tissue sections and in cultured keratocytes. **(C)** Cornea sections (left panel), as well as cultured central (middle panel) and peripheral (right panel) keratocytes were labeled with glutamate receptor NMDAR1 (TRITC; red) and double stained with DAPI. NMDAR1 was expressed in keratocytes in tissue sections and in cultured keratocytes. **(D)** 250,000 central or peripheral keratocytes grown in either 2% FBS or 0% FBS were lysed and expression of NMDAR1 (105 kDa) was analyzed by western blot. NMDAR1 expression was significantly higher in both central and peripheral keratocytes grown in 2% FBS than in central and peripheral keratocytes grown in 0% FBS (*p<0.05 for central keratocytes, *p<0.05 for peripheral keratocytes). Expression of NMDAR1 was also significantly higher in central keratocytes grown in 0% FBS than in peripheral keratocytes grown in same conditions (**p<0.01). β-actin (42 kDa) served as a loading control. Values are means ± SD.

Presence and differences in amount of glutamate was measured by glutamate assay. Two culture conditions were tested. Central and peripheral keratocytes were cultured in either DMEM/F12 medium supplemented with 2% FBS or 0% FBS. Central keratocytes grown in these two conditions, as well as peripheral keratocytes grown in these two conditions, were compared. Moreover, comparison was made between central and peripheral keratocytes. Levels of glutamate were significantly higher in central and peripheral keratocytes culture supernatants collected from cells grown in 0% FBS than in supernatants collected from cells grown in 2% FBS. Levels of glutamate in cell lysates were significantly higher in peripheral keratocytes grown in 2% FBS than in peripheral keratocytes grown in 0% FBS (Fig 5A). Expression of glutamate and its receptor NMDAR1 was also analyzed by immunohistochemistry and immunocytochemistry. Glutamate was expressed in both keratocytes in tissue sections and in cultured cells (Fig 5B). Glutamate receptor NMDAR1 was expressed in keratocytes in tissue sections and in cultured cells (Fig 5C). To quantify and compare NMDAR1 expression under different culturing conditions and two types of keratocytes, western blot and densitometry analyses were performed. NMDAR1 expression was significantly higher in both central and peripheral keratocytes grown in 2% FBS than in central and peripheral keratocytes grown in 0% FBS. Expression of NMDAR1 was also significantly higher in central keratocytes grown in 0% FBS than in peripheral keratocytes grown in the same condition.

## Discussion

The present study shows that human keratocytes express an array of neuropeptides and neurotransmitters that traditionally have been seen as neuron specific. It also shows that keratocytes express receptors for these substances, making them susceptible to stimulation by neuropeptides/neurotransmitters in an autocrine and/or paracrine manner, in the latter case possibly through secretion of substances from corneal nerves. The possible importance of this expression profile of neuropeptides and neurotransmitters in human corneal stromal cells is here discussed in the context of the known functional properties of these substances and their relevance for processes included in corneal wound healing.

### Methodological aspects: Phenotype of human keratocytes in primary cultures in vitro

In the absence of injury or pathology, keratocytes of the corneal stroma are considered as quiescent cells, with a low rate of proliferation and apoptosis [1]. However the culture conditions (including the factors added to the medium), the concentration of cells, and the stiffness of the support can influence the cell phenotype *in vitro* [34, 35]. For example, it has been shown that FBS can induce a fibroblastic phenotype on keratocytes [36]. To determine whether the culture conditions of the present study (2% FBS or 0% FBS) modified the cells’ phenotype and/or function, expression of several keratocyte markers was analyzed and compared between different culturing conditions. Keratocytes isolated from the stroma and cultured in serum free medium should retain their phenotype and maintain the expression of specific markers such as aldehyde dehydrogenase (ALDH) and keratocan. Keratocan, a cornea-specific keratan sulfate proteoglycan [31] was abundantly expressed in cultured cells regardless of the culturing condition (2% and 0% FBS). However, ALDH, a corneal crystalline which helps to maintain the cellular transparency was expressed in low amounts and surprisingly cells cultured in serum free medium expressed lower amounts of this marker. Lumican is a 38 kDa protein belonging to the small leucine-rich proteoglycans and is expressed in the extracellular matrix (ECM) of various tissues including the corneal stroma [37] in which it is produced by keratocytes. It has been well described that during activation of keratocytes to myofibroblasts the cells decrease the expression of keratan sulfate proteoglycans and increase the expression of α-SMA, a marker of myofibroblasts [38]. Under our culture conditions, cultured cells expressed high amounts of lumican, with non-significant differences between cells cultured in 2% and 0% FBS. CD34 is a well-established marker of quiescent keratocytes *in vivo*. Its role in keratocytes has not yet been elucidated but there is evidence that it might play a role in regulation of differentiation, adhesion, and quiescence. Under our culture conditions, cultured cells expressed high amounts of this protein. Cells cultured in serum free medium had lower expression of CD34 than cells cultured in 2% serum medium. Taken together, the cultured human corneal stroma cells of this study express the most important keratocyte markers and are thus deemed to be of a preserved keratocyte phenotype. Serum is able to alternate the cell phenotype, although to a minor degree in the amount of 2% FBS.

As the human cornea can be divided into a central and a limbal part (with partly different clinical features), we derived keratocytes from both parts separately to investigate if these two differ in expression patterns of neuropeptides/neurotransmitters. However, as precise separation of these two parts *in vitro* is impossible, we used a term ‘peripheral’ for keratocytes derived from the limbus region and adjacent corneal tissue. Both central and peripheral keratocytes expressed keratocan, which is a cornea-specific marker [7] that has been shown to be expressed in cultured keratocytes [6, 8–10].

### Neuropeptides and their receptors in keratocytes

The results of the present study show that both central and peripheral keratocytes in culture express the two tachykinins of interest, SP and NKA, as well as their preferred receptors NK-1R and NK-2R, respectively. SP and NKA belong to a family of neuropeptides which have a variety of pharmacological actions both in the central nervous system and in the periphery [28]. SP and its preferred receptor have been found to be expressed in tumor cells [39], tenocytes [29] and colonic epithelial cells [40], and also previously in cultured keratocytes from human cornea [18]. NKA expression has been discovered, for example, in monocytes and lymphocytes [41, 42]. These two substances have been linked to proliferative and apoptotic properties [43, 44], as well as being known to interact with components of extracellular matrix, mediating migration and playing a role in inflammatory responses [45]. As these mechanisms play a role in stromal wound healing, it might be speculated that both SP and NKA have effects in corneal wound healing processes. The fact that keratocytes also expressed the SP and NKA receptors, suggests that keratocytes themselves indeed could be subjected to SP and NKA actions.

### Catecholamines and their receptors in keratocytes

Presence of catecholamines (adrenaline, noradrenaline, and dopamine) was confirmed in both central and peripheral keratocytes in culture. It has been shown that catecholamines are synthesized both in the brain and in non-neuronal organs and cells such as adrenal medulla, gut cells, platelets, and lymphocytes [46–49]. However, when we analyzed expression of TH–an enzyme responsible for catalyzing the conversion of the amino acid L-tyrosine to L-DOPA–we found that cultured cells express it but the keratocytes in tissue sections do not. Moreover, when we analyzed the expression of adrenaline and noradrenaline adrenoreceptors (α1 adrenergic receptor [ADRA1] and β2 adrenergic receptor [ADRB2]) we found that they are not expressed in keratocytes in tissue sections, and that their expression in the cultured cells was inconclusive as their expression varied among cells obtained from different donors. As catecholamines play a role in stress response [50] it might be that the cultured cells are in stress conditions and that this explains why they sometimes express the adrenaline and noradrenaline receptors. Also, catecholamines have been found to regulate angiogenesis in tumors [51]. Taken together, the expression of catecholamines and the presence of adrenoreceptors in cultured cells but not in keratocytes in tissue sections suggest that they might play a role in corneal stress response, which might be due to injury.

### ACh and muscarinic receptors in keratocytes

ACh, a classical neurotransmitter, is thought to be synthesized by all living cells [30]. In the present study it was found that ACh is synthesized by both central and peripheral cultured keratocytes, and that these cells also express muscarinic ACh receptors (subtypes M_1_, M_3_, M_4_, and M_5_). Previous studies have shown that corneal epithelium contains high concentrations of ACh and its enzymes [19–21]. Furthermore, studies on mAChRs in corneal epithelium and endothelium have shown that mAChR M_2_, mAChR M_4_, and mAChR M_5_ are expressed by these cells, but that mAChR M_1_ and mAChR M_3_ are not [52]. It would be interesting to study why there are differences in the receptor patterns within the cornea and if there are any physiological reasons for that. ACh is known to induce proliferation in human tenocytes [53], and keratocytes [54] have an anti-apoptotic effect and facilitate wound healing in epithelial cell [55], as well as induce angiogenesis [56], all of which are effects that are of importance also in corneal wound healing.

### Glutamate and NMDA1R in keratocytes

Glutamate, an important neurotransmitter, and its receptors have been found in peripheral non-excitable cells in tissues such as taste buds, intestine, spleen, skin, and bone, as well as in platelets and lymphocytes [57–60]. In the present study, we found that cultured keratocytes express and secrete glutamate. Glutamate receptor NMDAR1 was also expressed by the keratocytes in culture. It has been found that glutamate is linked to modulation of tumor cell proliferation and migration [61], which might imply that it could play a similar role on cells in corneal would healing processes.

### Differences in expression profiles between peripheral and central keratocytes

Limbal epithelial stem cells are located at the basal epithelium of the palisades of Vogt, and regulated by their unique niche components [62]. Compared to the central keratocytes, the limbal keratocytes have a close spatial relationship with the limbal epithelial stem cells, suggesting the possibility of cytokine cross-talk between the two cell types. It has been reported that cells of limbal stromal origin contribute to the proliferation of limbal epithelial stem cells through paracrine signaling [63, 64]. Although limited reports describe the interactions between the limbal stem cells and other limbal niche components, the nerves may be involved in the regulation of limbal niche, since a previous study has confirmed that the limbal stroma is heavily innervated [65]. In the present study, we found that there were significant differences in the expression profiles of the neuropeptides/neurotransmitters and their receptors between peripheral and central corneal stromal cells, which might suggest possible differences in their contributions to the maintenance of limbal epithelial stem cells, a phenomenon previously shown [66].

### Concluding remarks

In conclusion, keratocytes in tissue sections and cultured human keratocytes express a broad range of neurotransmitters and neuropeptides as well as their receptors. The expression profiles seem to be changed upon culturing of the cells and show differences between peripheral and central keratocytes. The results, together with known effects of the neuropeptides/neurotransmitters in other tissues, suggest that neuronal and non-neuronal neuropeptides/neurotransmitters may play a role in corneal wound healing, which warrants further functional studies on their effect in different model systems of the cornea.

## Supporting Information

S1 Fig Gene expression analysis of neuropeptides and neurotransmitters. 250,000 cells were grown for 24h in 6 well plates. Cells were lysed and total mRNA was extracted. Gene expression was assessed by RT qPCR. Cultured central and peripheral keratocytes expressed substance P and neurokinin A gene (TAC1), genes involved in catecholamine synthesis (DDC), genes involved in glutamate synthesis (GLS, GOT), and receptor genes: TACR1 (for the substance P receptor NK-1R), TACR2 (for the neurokinin A receptor NK-2R), CHRM1-5 (muscarinic acetylcholine receptors M1-M5), DRD2 (dopamine receptor), ADRA1B and ADRB2 (adrenergic adrenaline and noradrenaline receptors).
(TIF)

## References

[1] West-Mays JA, Dwivedi DJ. The keratocyte: corneal stromal cell with variable repair phenotypes. The international journal of biochemistry & cell biology. 2006;38(10):1625–31. Epub 2006/05/06. 10.1016/j.biocel.2006.03.010 16675284 PMC2505273

[2] Beales MP, Funderburgh JL, Jester JV, Hassell JR. Proteoglycan synthesis by bovine keratocytes and corneal fibroblasts: maintenance of the keratocyte phenotype in culture. Investigative ophthalmology & visual science. 1999;40(8):1658–63. Epub 1999/07/07. . 10393032

[3] Robert L, Legeais JM, Robert AM, Renard G. Corneal collagens. Pathologie-biologie. 2001;49(4):353–63. Epub 2001/06/29. . 11428172 10.1016/s0369-8114(01)00144-4

[4] Hay ED. Development of the vertebrate cornea. International review of cytology. 1980;63:263–322. Epub 1979/01/01. . 395131 10.1016/s0074-7696(08)61760-x

[5] Muller LJ, Pels L, Vrensen GF. Novel aspects of the ultrastructural organization of human corneal keratocytes. Investigative ophthalmology & visual science. 1995;36(13):2557–67. Epub 1995/12/01. . 7499078

[6] Dahl IM. Biosynthesis of proteoglycans and hyaluronate in rabbit corneal fibroblast cultures. Variation with age of the cell line and effect of foetal calf serum. Experimental eye research. 1981;32(4):419–33. Epub 1981/04/01. . 7238627 10.1016/s0014-4835(81)80021-8

[7] Midura RJ, Hascall VC. Analysis of the proteoglycans synthesized by corneal explants from embryonic chicken. II. Structural characterization of the keratan sulfate and dermatan sulfate proteoglycans from corneal stroma. The Journal of biological chemistry. 1989;264(3):1423–30. Epub 1989/01/25. . 2521480

[8] Funderburgh JL, Funderburgh ML, Mann MM, Prakash S, Conrad GW. Synthesis of corneal keratan sulfate proteoglycans by bovine keratocytes in vitro. The Journal of biological chemistry. 1996;271(49):31431–6. Epub 1996/12/06. . 8940154 10.1074/jbc.271.49.31431

[9] Hassell JR, Schrecengost PK, Rada JA, SundarRaj N, Sossi G, Thoft RA. Biosynthesis of stromal matrix proteoglycans and basement membrane components by human corneal fibroblasts. Investigative ophthalmology & visual science. 1992;33(3):547–57. Epub 1992/03/01. . 1544783

[10] Yue BY, Baum JL. The synthesis of glycosaminoglycans by cultures of rabbit corneal endothelial and stromal cells. The Biochemical journal. 1976;158(3):567–73. Epub 1976/09/15. 985449 10.1042/bj1580567 PMC1164011

[11] Masur SK, Cheung JK, Antohi S. Identification of integrins in cultured corneal fibroblasts and in isolated keratocytes. Investigative ophthalmology & visual science. 1993;34(9):2690–8. Epub 1993/08/01. . 8344791

[12] Masur SK, Dewal HS, Dinh TT, Erenburg I, Petridou S. Myofibroblasts differentiate from fibroblasts when plated at low density. Proceedings of the National Academy of Sciences of the United States of America. 1996;93(9):4219–23. Epub 1996/04/30. 8633044 10.1073/pnas.93.9.4219 PMC39515

[13] Jester JV, Barry PA, Lind GJ, Petroll WM, Garana R, Cavanagh HD. Corneal keratocytes: in situ and in vitro organization of cytoskeletal contractile proteins. Investigative ophthalmology & visual science. 1994;35(2):730–43. Epub 1994/02/01. . 8113024

[14] Jester JV, Barry-Lane PA, Cavanagh HD, Petroll WM. Induction of alpha-smooth muscle actin expression and myofibroblast transformation in cultured corneal keratocytes. Cornea. 1996;15(5):505–16. Epub 1996/09/01. . 8862928

[15] Wilson SE, Mohan RR, Mohan RR, Ambrosio R Jr., Hong J, Lee J. The corneal wound healing response: cytokine-mediated interaction of the epithelium, stroma, and inflammatory cells. Progress in retinal and eye research. 2001;20(5):625–37. Epub 2001/07/27. . 11470453 10.1016/s1350-9462(01)00008-8

[16] Fini ME, Stramer BM. How the cornea heals: cornea-specific repair mechanisms affecting surgical outcomes. Cornea. 2005;24(8 Suppl):S2–S11. Epub 2005/10/18. . 16227819 10.1097/01.ico.0000178743.06340.2c

[17] Netto MV, Mohan RR, Ambrosio R Jr., Hutcheon AE, Zieske JD, Wilson SE. Wound healing in the cornea: a review of refractive surgery complications and new prospects for therapy. Cornea. 2005;24(5):509–22. Epub 2005/06/22. . 15968154 10.1097/01.ico.0000151544.23360.17

[18] Watanabe M, Nakayasu K, Iwatsu M, Kanai A. Endogenous substance P in corneal epithelial cells and keratocytes. Japanese journal of ophthalmology. 2002;46(6):616–20. Epub 2003/01/25. . 12543186 10.1016/s0021-5155(02)00617-2

[19] Gnadinger MC, Heimann R, Markstein R. Choline acetyltransferase in corneal epithilium. Experimental eye research. 1973;15(3):395–9. Epub 1973/03/01. . 4695443 10.1016/0014-4835(73)90155-3

[20] Mindel JS, Mittag TW. Choline acetyltransferase in ocular tissues of rabbits, cats, cattle, and man. Investigative ophthalmology. 1976;15(10):808–14. Epub 1976/10/01. . 977252

[21] Van Alphen GW. Acetylcholine synthesis in corneal epithelium. AMA archives of ophthalmology. 1957;58(3):449–51. Epub 1957/09/01. . 13457539 10.1001/archopht.1957.00940010461022

[22] Muller LJ, Marfurt CF, Kruse F, Tervo TM. Corneal nerves: structure, contents and function. Experimental eye research. 2003;76(5):521–42. Epub 2003/04/17. . 12697417 10.1016/s0014-4835(03)00050-2

[23] Pradhan L, Nabzdyk C, Andersen ND, LoGerfo FW, Veves A. Inflammation and neuropeptides: the connection in diabetic wound healing. Expert reviews in molecular medicine. 2009;11:e2 Epub 2009/01/14. 10.1017/S1462399409000945 19138453 PMC3708299

[24] Brain SD. Sensory neuropeptides: their role in inflammation and wound healing. Immunopharmacology. 1997;37(2–3):133–52. Epub 1997/12/24. . 9403332 10.1016/s0162-3109(97)00055-6

[25] Blais M, Mottier L, Germain MA, Bellenfant S, Cadau S, Berthod F. Sensory Neurons Accelerate Skin Reepithelialization via Substance P in an Innervated Tissue-Engineered Wound Healing Model. Tissue engineering Part A. 2014;20(15–16):2180–8. Epub 2014/04/11. 10.1089/ten.tea.2013.0535 24716723 PMC4137331

[26] Cheret J, Lebonvallet N, Carre JL, Misery L, Le Gall-Ianotto C. Role of neuropeptides, neurotrophins, and neurohormones in skin wound healing. Wound repair and regeneration: official publication of the Wound Healing Society [and] the European Tissue Repair Society. 2013;21(6):772–88. Epub 2013/10/19. 10.1111/wrr.12101 . 24134750

[27] Cheret J, Lebonvallet N, Buhe V, Carre JL, Misery L, Le Gall-Ianotto C. Influence of sensory neuropeptides on human cutaneous wound healing process. Journal of dermatological science. 2014;74(3):193–203. Epub 2014/03/19. 10.1016/j.jdermsci.2014.02.001 . 24630238

[28] Nelson DA, Bost KL. Non-neuronal mammalian tachykinin expression. Frontiers in bioscience: a journal and virtual library. 2004;9:2166–76. Epub 2004/09/09. . 15353278 10.2741/1372

[29] Andersson G, Danielson P, Alfredson H, Forsgren S. Presence of substance P and the neurokinin-1 receptor in tenocytes of the human Achilles tendon. Regulatory peptides. 2008;150(1–3):81–7. Epub 2008/04/09. 10.1016/j.regpep.2008.02.005 . 18394729

[30] Wessler I, Kirkpatrick CJ. Acetylcholine beyond neurons: the non-neuronal cholinergic system in humans. British journal of pharmacology. 2008;154(8):1558–71. Epub 2008/05/27. 10.1038/bjp.2008.185 18500366 PMC2518461

[31] Carlson EC, Liu CY, Chikama T, Hayashi Y, Kao CW, Birk DE, et al Keratocan, a cornea-specific keratan sulfate proteoglycan, is regulated by lumican. The Journal of biological chemistry. 2005;280(27):25541–7. Epub 2005/04/26. 10.1074/jbc.M500249200 15849191 PMC2874675

[32] Sidney LE, Branch MJ, Dunphy SE, Dua HS, Hopkinson A. Concise review: evidence for CD34 as a common marker for diverse progenitors. Stem cells. 2014;32(6):1380–9. Epub 2014/02/06. 10.1002/stem.1661 24497003 PMC4260088

[33] Musselmann K, Alexandrou B, Kane B, Hassell JR. Maintenance of the keratocyte phenotype during cell proliferation stimulated by insulin. The Journal of biological chemistry. 2005;280(38):32634–9. Epub 2005/09/20. 10.1074/jbc.M504724200 . 16169858

[34] Ahearne M, Wilson SL, Liu KK, Rauz S, El Haj AJ, Yang Y. Influence of cell and collagen concentration on the cell-matrix mechanical relationship in a corneal stroma wound healing model. Experimental eye research. 2010;91(5):584–91. Epub 2010/08/04. 10.1016/j.exer.2010.07.013 . 20678499

[35] Lakshman N, Petroll WM. Growth factor regulation of corneal keratocyte mechanical phenotypes in 3-D collagen matrices. Investigative ophthalmology & visual science. 2012;53(3):1077–86. Epub 2012/01/17. 10.1167/iovs.11-8609 22247479 PMC3339898

[36] Kim A, Zhou C, Lakshman N, Petroll WM. Corneal stromal cells use both high- and low-contractility migration mechanisms in 3-D collagen matrices. Experimental cell research. 2012;318(6):741–52. Epub 2012/01/12. 10.1016/j.yexcr.2011.12.018 22233682 PMC3302352

[37] Hassell JR, Newsome DA, Krachmer JH, Rodrigues MM. Macular corneal dystrophy: failure to synthesize a mature keratan sulfate proteoglycan. Proceedings of the National Academy of Sciences of the United States of America. 1980;77(6):3705–9. Epub 1980/06/01. 6447876 10.1073/pnas.77.6.3705 PMC349687

[38] Jester JV, Petroll WM, Barry PA, Cavanagh HD. Expression of alpha-smooth muscle (alpha-SM) actin during corneal stromal wound healing. Investigative ophthalmology & visual science. 1995;36(5):809–19. Epub 1995/04/01. . 7706029

[39] Munoz M, Covenas R. Neurokinin-1 receptor: a new promising target in the treatment of cancer. Discovery medicine. 2010;10(53):305–13. Epub 2010/11/03. . 21034671

[40] Zhao D, Kuhnt-Moore S, Zeng H, Pan A, Wu JS, Simeonidis S, et al Substance P-stimulated interleukin-8 expression in human colonic epithelial cells involves Rho family small GTPases. The Biochemical journal. 2002;368(Pt 2):665–72. Epub 2002/08/10. 10.1042/BJ20020950 12169092 PMC1222994

[41] Ho WZ, Lai JP, Zhu XH, Uvaydova M, Douglas SD. Human monocytes and macrophages express substance P and neurokinin-1 receptor. Journal of immunology. 1997;159(11):5654–60. Epub 1998/04/21. . 9548509

[42] Lai JP, Douglas SD, Ho WZ. Human lymphocytes express substance P and its receptor. Journal of neuroimmunology. 1998;86(1):80–6. Epub 1998/07/09. . 9655475 10.1016/s0165-5728(98)00025-3

[43] Backman LJ, Fong G, Andersson G, Scott A, Danielson P. Substance P is a mechanoresponsive, autocrine regulator of human tenocyte proliferation. PloS one. 2011;6(11):e27209 Epub 2011/11/10. 10.1371/journal.pone.0027209 22069500 PMC3206074

[44] Backman LJ, Andersson G, Fong G, Alfredson H, Scott A, Danielson P. Alpha-2 adrenergic stimulation triggers Achilles tenocyte hypercellularity: Comparison between two model systems. Scandinavian journal of medicine & science in sports. 2013;23(6):687–96. Epub 2012/02/02. 10.1111/j.1600-0838.2011.01442.x . 22292987 PMC3933766

[45] Levite M, Cahalon L, Hershkoviz R, Steinman L, Lider O. Neuropeptides, via specific receptors, regulate T cell adhesion to fibronectin. Journal of immunology. 1998;160(2):993–1000. Epub 1998/04/29. . 9551939

[46] Eisenhofer G, Aneman A, Hooper D, Rundqvist B, Friberg P. Mesenteric organ production, hepatic metabolism, and renal elimination of norepinephrine and its metabolites in humans. Journal of neurochemistry. 1996;66(4):1565–73. Epub 1996/04/01. . 8627312 10.1046/j.1471-4159.1996.66041565.x

[47] Eisenhofer G, Aneman A, Friberg P, Hooper D, Fandriks L, Lonroth H, et al Substantial production of dopamine in the human gastrointestinal tract. The Journal of clinical endocrinology and metabolism. 1997;82(11):3864–71. Epub 1997/11/14. 10.1210/jcem.82.11.4339 . 9360553

[48] Goldstein DS, Eisenhofer G, Kopin IJ. Sources and significance of plasma levels of catechols and their metabolites in humans. The Journal of pharmacology and experimental therapeutics. 2003;305(3):800–11. Epub 2003/03/22. 10.1124/jpet.103.049270 . 12649306

[49] Basu S, Dasgupta PS. Dopamine, a neurotransmitter, influences the immune system. Journal of neuroimmunology. 2000;102(2):113–24. Epub 2000/01/15. . 10636479 10.1016/s0165-5728(99)00176-9

[50] Glaser R, Kiecolt-Glaser JK. Stress-induced immune dysfunction: implications for health. Nature reviews Immunology. 2005;5(3):243–51. Epub 2005/03/02. 10.1038/nri1571 . 15738954

[51] Sarkar C, Chakroborty D, Basu S. Neurotransmitters as regulators of tumor angiogenesis and immunity: the role of catecholamines. Journal of neuroimmune pharmacology: the official journal of the Society on NeuroImmune Pharmacology. 2013;8(1):7–14. Epub 2012/08/14. 10.1007/s11481-012-9395-7 22886869 PMC3869381

[52] Grueb M, Reinthal E, Rohrbach JM, Bartz-Schmidt KU. Muscarinic acetylcholine receptor subtypes in human corneal epithelium and endothelium. Graefe's archive for clinical and experimental ophthalmology = Albrecht von Graefes Archiv fur klinische und experimentelle Ophthalmologie. 2006;244(9):1191–5. Epub 2006/03/01. 10.1007/s00417-006-0263-0 . 16506072

[53] Fong G, Backman LJ, Andersson G, Scott A, Danielson P. Human tenocytes are stimulated to proliferate by acetylcholine through an EGFR signalling pathway. Cell and tissue research. 2013;351(3):465–75. Epub 2012/12/06. 10.1007/s00441-012-1530-5 23212463 PMC3582816

[54] Sloniecka M, Backman LJ, Danielson P. Acetylcholine enhances keratocyte proliferation through muscarinic receptor activation. International immunopharmacology. 2015 Epub 2015/06/07. 10.1016/j.intimp.2015.05.039 . 26049030

[55] Grando SA, Pittelkow MR, Schallreuter KU. Adrenergic and cholinergic control in the biology of epidermis: physiological and clinical significance. The Journal of investigative dermatology. 2006;126(9):1948–65. Epub 2006/08/17. 10.1038/sj.jid.5700151 . 16912692

[56] Cooke JP. Angiogenesis and the role of the endothelial nicotinic acetylcholine receptor. Life sciences. 2007;80(24–25):2347–51. Epub 2007/03/27. 10.1016/j.lfs.2007.01.061 17383685 PMC1941778

[57] Gill SS, Pulido OM. Glutamate receptors in peripheral tissues: current knowledge, future research, and implications for toxicology. Toxicologic pathology. 2001;29(2):208–23. Epub 2001/06/26. . 11421488 10.1080/019262301317052486

[58] Nicoletti F, Battaglia G, Storto M, Ngomba RT, Iacovelli L, Arcella A, et al Metabotropic glutamate receptors: beyond the regulation of synaptic transmission. Psychoneuroendocrinology. 2007;32 Suppl 1:S40–5. Epub 2007/07/27. 10.1016/j.psyneuen.2007.04.015 . 17651904

[59] Haas HS, Linecker A, Pfragner R, Sadjak A. Peripheral glutamate signaling in head and neck areas. Head & neck. 2010;32(11):1554–72. Epub 2010/09/18. 10.1002/hed.21438 . 20848447

[60] Julio-Pieper M, Flor PJ, Dinan TG, Cryan JF. Exciting times beyond the brain: metabotropic glutamate receptors in peripheral and non-neural tissues. Pharmacological reviews. 2011;63(1):35–58. Epub 2011/01/14. 10.1124/pr.110.004036 . 21228260

[61] Haas HS, Pfragner R, Tabrizi-Wizsy NG, Rohrer K, Lueftenegger I, Horwath C, et al The influence of glutamate receptors on proliferation and metabolic cell activity of neuroendocrine tumors. Anticancer research. 2013;33(4):1267–72. Epub 2013/04/09. . 23564764

[62] Li W, Hayashida Y, Chen YT, Tseng SC. Niche regulation of corneal epithelial stem cells at the limbus. Cell research. 2007;17(1):26–36. Epub 2007/01/11. 10.1038/sj.cr.7310137 17211449 PMC3190132

[63] Notara M, Shortt AJ, Galatowicz G, Calder V, Daniels JT. IL6 and the human limbal stem cell niche: a mediator of epithelial-stromal interaction. Stem cell research. 2010;5(3):188–200. Epub 2010/09/04. 10.1016/j.scr.2010.07.002 . 20813601

[64] Gonzalez S, Deng SX. Presence of native limbal stromal cells increases the expansion efficiency of limbal stem/progenitor cells in culture. Experimental eye research. 2013;116:169–76. Epub 2013/09/11. 10.1016/j.exer.2013.08.020 24016868 PMC3900305

[65] Lawrenson JG, Ruskell GL. The structure of corpuscular nerve endings in the limbal conjunctiva of the human eye. Journal of anatomy. 1991;177:75–84. Epub 1991/08/01. 1769901 PMC1260416

[66] Ainscough SL, Linn ML, Barnard Z, Schwab IR, Harkin DG. Effects of fibroblast origin and phenotype on the proliferative potential of limbal epithelial progenitor cells. Experimental eye research. 2011;92(1):10–9. Epub 2010/10/26. 10.1016/j.exer.2010.10.004 . 20970420

