# Supplementary figures and images for: Expression Profiles of Neuropeptides, Neurotransmitters, and Their Receptors in Human Keratocytes In Vitro and In Situ
Source: PLoS One. 2015 Jul 27;10(7):e0134157. doi: 10.1371/journal.pone.0134157 (PMC4516240; doi:10.1371/journal.pone.0134157)

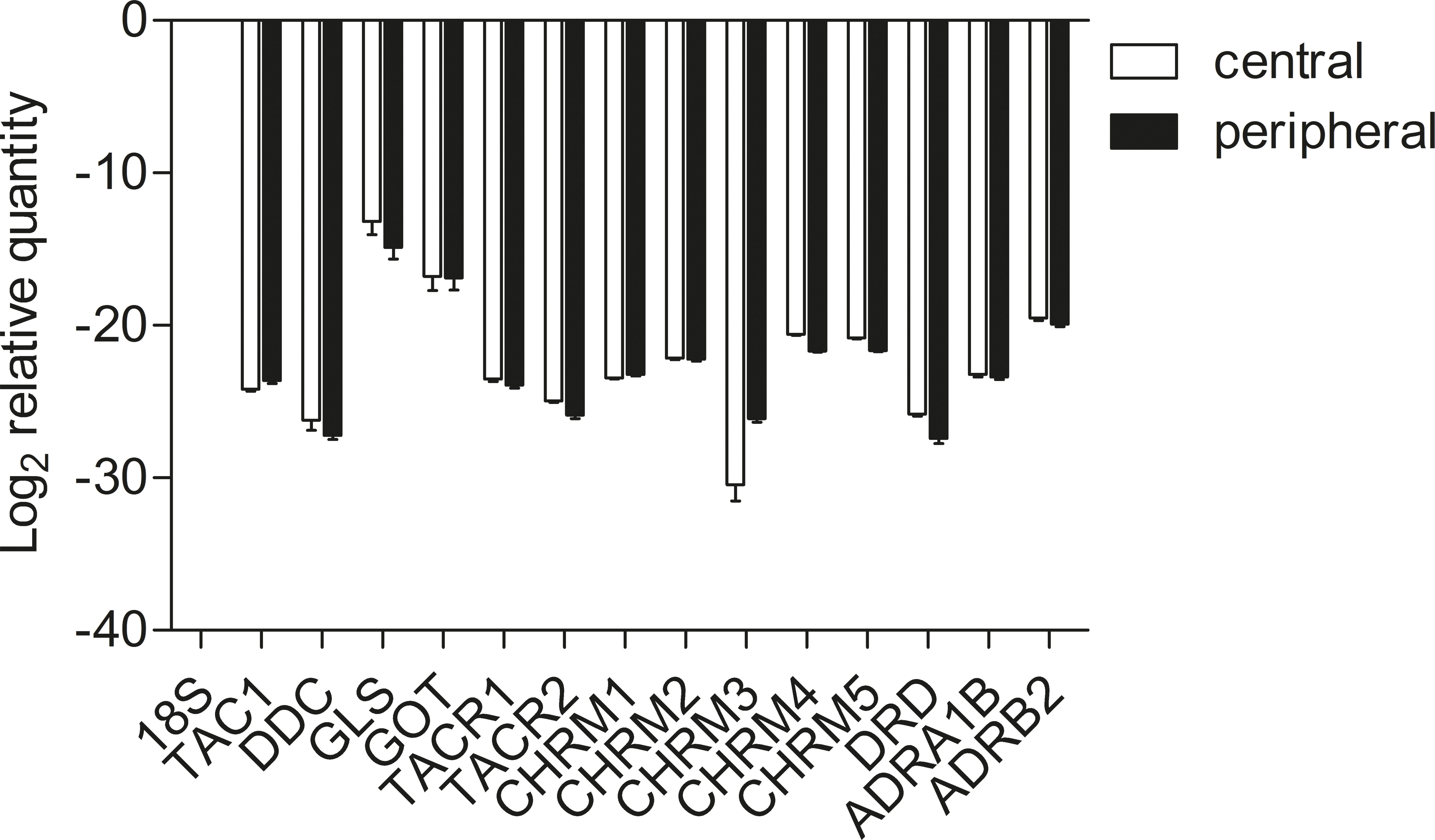

Supplement: S1 Fig — 250,000 cells were grown for 24h in 6 well plates. Cells were lysed and total mRNA was extracted. Gene expression was assessed by RT qPCR. Cultured central and peripheral keratocytes expressed substance P and neurokinin A gene (TAC1), genes involved in catecholamine synthesis (DDC), genes involved in glutamate synthesis (GLS, GOT), and receptor genes: TACR1 (for the substance P receptor NK-1R), TACR2 (for the neurokinin A receptor NK-2R), CHRM1-5 (muscarinic acetylcholine receptors M1-M5), DRD2 (dopamine receptor), ADRA1B and ADRB2 (adrenergic adrenaline and noradrenaline receptors). (TIF) [file pone.0134157.s001.tif]
